# A 3D culture system for evaluating the combined effects of cisplatin and anti-fibrotic drugs on the growth and invasion of lung cancer cells co-cultured with fibroblasts

**DOI:** 10.1063/5.0115464

**Published:** 2023-03-28

**Authors:** Huei-Jyuan Pan, Chia-Wei Lee, Li-Yu Wu, Heng-Hua Hsu, Yi-Chung Tung, Wei-Yu Liao, Chau-Hwang Lee

**Affiliations:** 1 Research Center for Applied Sciences, Academia Sinica, Taipei 11529, Taiwan; 2 Institute of Biophotonics, National Yang Ming Chiao Tung University, Taipei 11221, Taiwan; 3 Department of Internal Medicine, National Taiwan University Hospital, Taipei 10002, Taiwan; 4 Department of Internal Medicine, College of Medicine, National Taiwan University, Taipei 10002, Taiwan

## Abstract

Fibrosis and fibroblast activation usually occur in the tissues surrounding a malignant tumor; therefore, anti-fibrotic drugs are used in addition to chemotherapy. A reliable technique for evaluating the combined effects of anti-fibrotic drugs and anticancer drugs would be beneficial for the development of an appropriate treatment strategy. In this study, we manufactured a three-dimensional (3D) co-culture system of fibroblasts and lung cancer cell spheroids in Matrigel supplemented with fibrin (fibrin/Matrigel) that simulated the tissue microenvironment around a solid tumor. We compared the efficacy of an anticancer drug (cisplatin) with or without pretreatments of two anti-fibrotic drugs, nintedanib and pirfenidone, on the growth and invasion of cancer cells co-cultured with fibroblasts. The results showed that the addition of nintedanib improved cisplatin's effects on suppressing the growth of cancer cell spheroids and the invasion of cancer cells. In contrast, pirfenidone did not enhance the anticancer activity of cisplatin. Nintedanib also showed higher efficacy than pirfenidone in reducing the expression of four genes in fibroblasts associated with cell adhesion, invasion, and extracellular matrix degradation. This study demonstrated that the 3D co-cultures in fibrin/Matrigel would be useful for assessing the effects of drug combinations on tumor growth and invasion.

## INTRODUCTION

The extracellular matrix (ECM) surrounding a solid tumor plays important roles in tumor progression and therapeutic efficacy.^1,2^ In the tumor microenvironment (TME), cancer-associated fibroblasts (CAFs) facilitate the production of various cytokines in favor of cancer cell proliferation and metastasis.^3–7^ CAFs also alter the ECM's properties and affect tumor progression and therapy resistance.^8^ Consequently, CAFs are important therapeutic targets in anticancer treatments. However, assessing the effect of ECM modulations on anticancer drug effects *in vivo* is difficult because it requires advanced imaging techniques to evaluate the properties of ECM, CAFs, and cancer cell viability simultaneously in the TME inside a live animal.^9^ Therefore, an *in vitro* model with a satisfactory predictive capacity is highly desirable for evaluating the efficacy of antitumor therapies.

Three-dimensional (3D) cell culture technology has provided new opportunities for observing and analyzing cell activities in artificial environments close to real tissues. Hydrogels made of natural or synthetic biomaterials containing ECM components such as collagen, laminin, and polysaccharides are used to construct 3D culture models. For example, cellular spheroids consisting of cancer cells resemble solid tumors, whereas the surrounding ECM and co-cultured stromal cells constitute the TME.^10–12^ Three-dimensional cell cultures are used in studies on angiogenesis,^13^ cancer invasion,^14–17^ and ECM variations.^18–21^ In addition, various types of cells, such as fibroblasts and macrophages, produce cytokines and proteins, including matrix metalloproteinases (MMP), collagen, and fibronectin, which directly modulate the properties of ECM.^22^ Therefore, 3D co-culture models exhibit a great potential for testing drugs that change the ECM's properties combined with other types of drugs.

The physical and chemical properties of culture hydrogels are essential in 3D cell experiments and drug screen applications.^23^ Although Matrigel has been widely used in 3D cell experiments, Matrigel alone may not be useful for the assessment of cell invasion. For example, Guzman *et al.* reported that without the use of fibrillar collagen, the invasion of breast cancer cells in Matrigel was highly suppressed.^24^ Fibrin-based hydrogels were recently used as 3D endothelial cell network-formation platforms.^25,26^ In the present study, we followed the report of Broguiere *et al.* to improve the physical support of Matrigel by fibrin^27^ and built a 3D environment suitable for assessing the invasion of cancer cells co-cultured with fibroblasts. We used focused ion beam–scanning electron microscopy (FIB-SEM) imaging and atomic force microscopy (AFM) to characterize the microscopic features of the mixture of fibrin and Matrigel (fibrin/Matrigel). Using this 3D co-culture system, we compared the effects of two anti-fibrotic drugs, nintedanib (NTD) and pirfenidone (PFD), on the efficacy of cisplatin (CDDP) to inhibit the growth of tumor and suppress the invasion of cancer cells.

## RESULTS AND DISCUSSION

### Characterization of Matrigel, fibrin, and fibrin/Matrigel

**FIG. 1.**
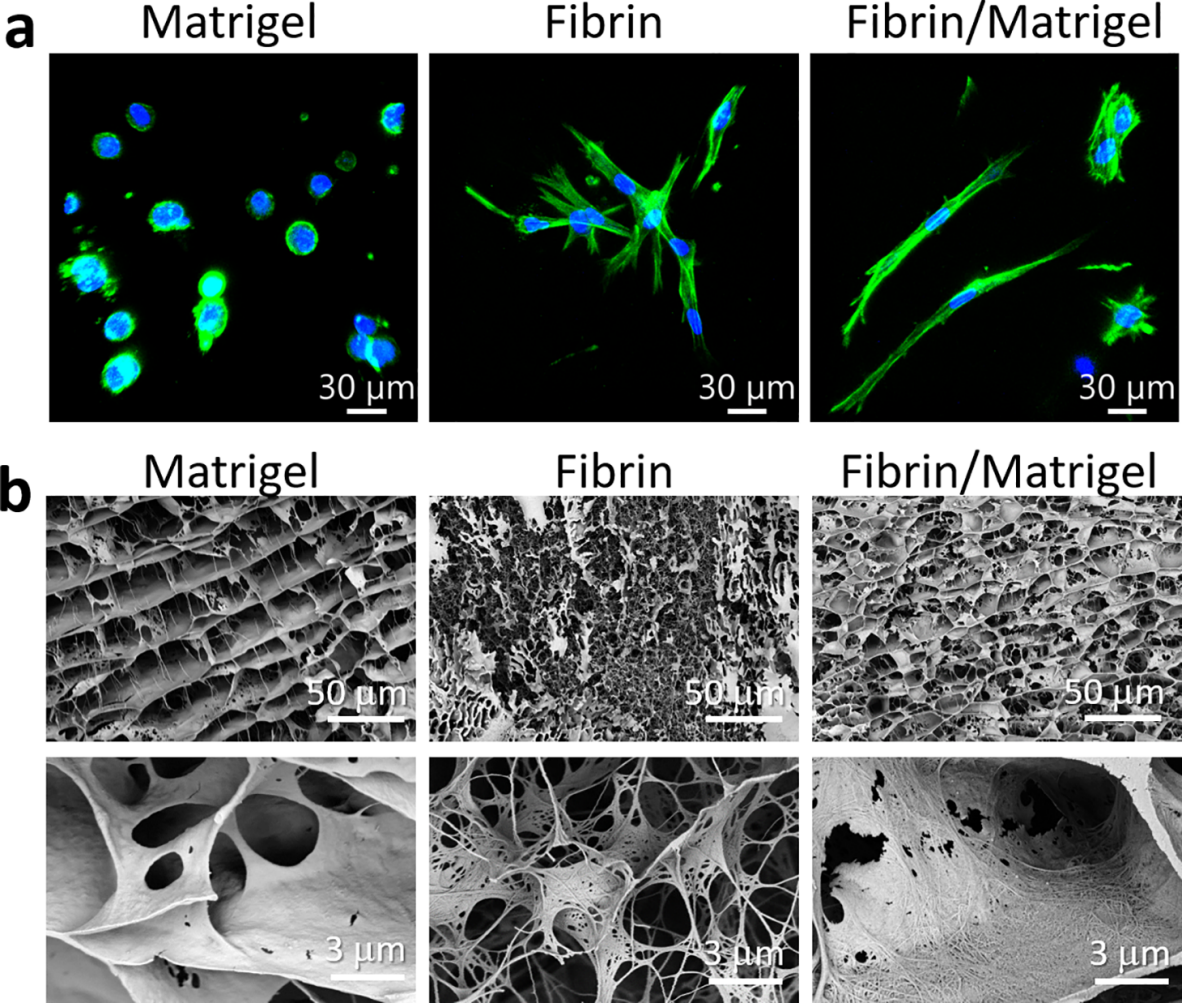
Morphology of MRC-5 fibroblasts and fine structures in the three types of hydrogels. (a) Confocal fluorescence projection images of fixed MRC-5 fibroblasts in hydrogels after the 24-h culture. Green, F-actin labeled with Alexa Fluor™ 488 Phalloidin. Blue, cell nuclei labeled with DAPI. (b) FIB-SEM images of the three types of hydrogels with two magnifications. The microstructures of the three types of hydrogels are different: Matrigel mostly consists of sheet structures, whereas fibrin mostly consists of fibrillar structures. In fibrin/Matrigel, the fibrin fiber was adhered to the surface of Matrigel sheets.

To evaluate whether the fibrin/Matrigel is suitable for 3D invasion assays, we first cultured MRC-5 fibroblasts in Matrigel, fibrin, and fibrin/Matrigel. As shown in Fig. 1(a), the fibroblasts in 3 mg/ml Matrigel became rounded with F-actin surrounding the nucleus. Fibroblasts would not migrate with such a morphology. In contrast, in 5 mg/mL fibrin and the fibrin/Matrigel, fibroblasts extended into a spindle shape with elongated F-actin. We speculated that the stiffness of 3 mg/ml Matrigel might be too low for the attachment and extension of fibroblasts.^28^ Moreover, the gene profiles of cells cultured in fibrin or fibrin/Matrigel might be different from those of cells in Matrigel.^29^ Because pure fibrin might not resemble the ECM constituents in the TME, we decided to use fibrin/Matrigel in the following experiments.

We used FIB-SEM to observe the microstructures of these hydrogels. The micrographs in Fig. 1(b) show that Matrigel exhibited obvious sheet structures, whereas fibrin formed mostly fibrillar structures. We measured the average pore sizes of Matrigel, fibrin, and fibrin/Matrigel in the SEM images to be 2.06 ± 1.36, 0.79 ± 0.29, and 1.34 ± 0.87 *μ*m^2^, respectively. The average pore densities of Matrigel, fibrin, and fibrin/Matrigel were 0.09 ± 0.04, 0.56 ± 0.17, and 0.23 ± 0.08 *μ*m^−2^, respectively. Compared with Matrigel, the addition of fibrin slightly reduced the pore size and increased the pore density. We found that in fibrin/Matrigel, the fibrin fiber was adhered to the surface of the Matrigel sheet structures. The fibrillar structures of fibrin and fibrin/Matrigel could also be necessary for 3D cancer cell invasion, as proposed by Berger *et al.*^30^

AFM analysis showed that the mean stiffness values of 3 mg/ml Matrigel and 5 mg/ml fibrin were 6 ± 2 and 258 ± 25 Pa, respectively, whereas that of fibrin/Matrigel was 105 ± 44 Pa. The stiffness distributions in each type of hydrogel are shown in supplementary material Fig. S1. The AFM analysis suggested that the fibrillar structures of fibrin/Matrigel shown in Fig. 1(b) provided additional physical support to the hydrogel.

We also performed the alamarBlue assay to quantify the viabilities of A549 cancer cells and MRC-5 fibroblasts in the three types of hydrogels. The data in supplementary material Fig. S2(a) show that both types of cells preserved high viabilities in these hydrogels. The invasiveness of cancer cells and fibroblasts quantified by the transwell assay is shown in supplementary material Fig. S2(b). The invasion capabilities of monocultured cancer cells or fibroblasts were similar in the three types of hydrogels.

**FIG. 2.**
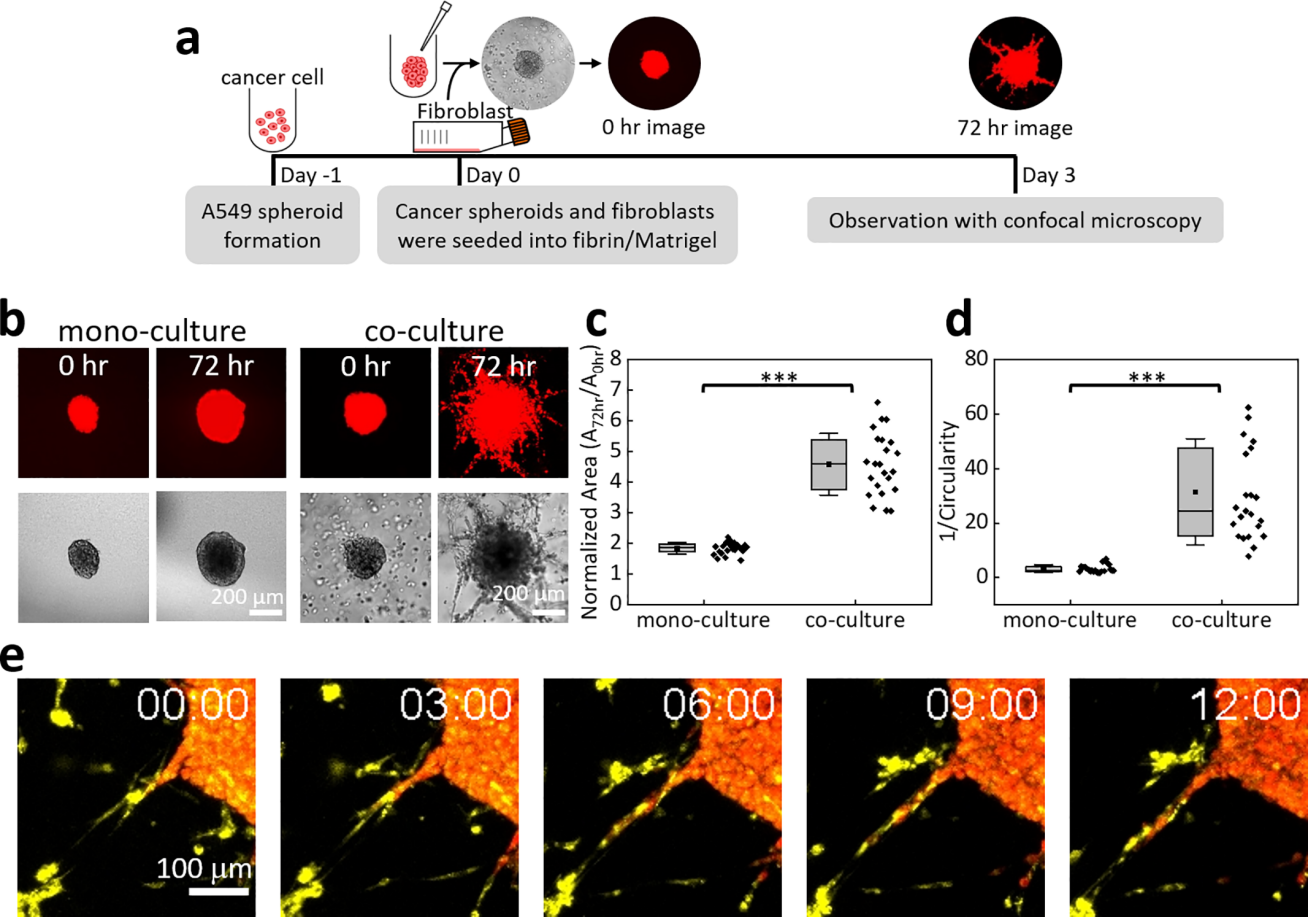
Variations of A549 cancer cell spheroids in fibrin/Matrigel caused by MRC-5 fibroblasts. (a) Experimental procedures for cancer cell spheroids co-cultured with fibroblasts. Only cancer cells were loaded with CellTracker™ Red CMTPX Dye. (b) Confocal fluorescence projection (top) and bright-field (bottom) images of typical A549 cell spheroids in fibrin/Matrigel without and with fibroblast co-culture. (c) Projection areas and (d) 1/Circularity values of A549 cell spheroids without and with fibroblast co-culture after 72 h. The data in (c) and (d) were from four independent experiments. Each dot represents one spheroid. ***, *P* < 0.001 (Mann–Whitney U test). (e) Time-lapse confocal microscopy images of cancer cells (red) and fibroblasts (yellow) near the rim of a cancer cell spheroid within 12 h of observation. Multimedia view: https://doi.org/10.1063/5.0115464.1
10.1063/5.0115464.1

Collagen is widely used in various hydrogels for 3D cell or tissue culture.^11^ Nonetheless, if fibroblasts exist in the culture system, the amounts of collagen produced by the fibroblasts could be a major variable reflecting the activity of fibroblasts; therefore, the exogenous collagen may be considered an undesirable background factor. In addition, myofibroblasts could exert deformation forces along fibrillar collagen ECM up to hundreds of micrometers.^31^ ECM deformation could affect the migration and invasion of nearby cells. Considering these points, we suggest that the fibrin/Matrigel used in the present study should be more suitable for assessing 3D cancer cell invasion under the effect of fibroblasts. Moreover, although the properties of Matrigel could vary from batch to batch, at least stiffness could be controlled by adjusting the amount of fibrin in fibrin/Matrigel.

### Cancer cell spheroids in fibrin/Matrigel without and with fibroblasts

In the co-culture experiments, we cultured one cancer cell spheroid with fibroblasts in a well containing fibrin/Matrigel and then observed the cell spheroids at 0 h and 72 h via confocal microscopy [Fig. 2(a)]. Figure 2(b) shows the intensity projection fluorescence and bright-field images of cancer cell spheroids in fibrin/Matrigel with and without the co-culture of MRC-5 fibroblasts. In these images, only the cancer cells were loaded with the fluorescent dye. Compared with our previous results on cancer cell spheroids and fibroblasts in Matrigel,^16^ the images in Fig. 2(b) showed much more obvious cancer cell invasion with the co-culture of fibroblasts, demonstrating better potential of fibrin/Matrigel for spheroid invasion assays.

In Fig. 2(c), the mean projection area of monoculture cancer cell spheroids at 72 h normalized to the initial value was 1.8 ± 0.2. We controlled the initial cancer cell numbers to be ∼1000 per well. The coefficient of variation of spheroid areas at 0 h for 327 monoculture spheroids was 18.4%. With the co-culture of fibroblasts (concentration: 1 × 10^6^ ml^−1^) for 72 h, the normalized spheroid area was 4.6 ± 1.0, i.e., 2.5-fold of that in the monoculture. As an evaluation of the invasiveness of the cancer cells, we used the inverse of spheroid circularity (1/*C* value) to quantify the irregularity of spheroid circumferences. Higher cell invasion resulted in more protrusions of the spheroid, which increased the 1/*C* value. For monocultured spheroids, the average 1/*C* value after 72 h in fibrin/Matrigel was 3.2 ± 1.4. The co-culture with fibroblasts significantly increased the average 1/*C* value to 31.5 ± 19.5, indicating more cancer cell invasion in the co-culture [Fig. 2(d)]. This result agrees with the well-known fibroblast-assisted cancer cell invasion.^4,5,7,32,33^ The major pro-invasion factors produced by fibroblasts include the hepatocyte growth factor, interleukin-6, transforming growth factor-β, and matrix metallopeptidases (MMPs). On the other hand, fibroblasts or cancer cells might produce collagen and other proteins to modify the structure and stiffness of the ECM, which could affect the cancer cell invasion and progression with complicated mechanisms.^34,35^ Because higher stiffness could accompany a denser fibrillar network, higher matrix stiffness does not necessarily promote cell invasion.^36^ To reveal how the ECM's mechanical properties influence cancer cell invasion under the effects from co-cultured fibroblasts, more experimental work and numerical simulations are required.

Another mechanism by which co-cultured fibroblasts improve cancer cell invasiveness could be fibroblast-led invasion.^15,16,37–39^ Figure 2(e) (Multimedia view) shows time-lapse confocal microscopy images of the region near the rim of an A549 cell spheroid. Fibroblasts (yellow) led the invasion of A549 cells (red) into the fibrin/Matrigel. This result confirmed that fibroblast-led invasion also played a role in fibroblast-enhanced cancer cell invasion in our co-culture system.

### Effects of anti-fibrotic drugs on cancer cell spheroids co-cultured with fibroblasts in fibrin/Matrigel

**FIG. 3.**
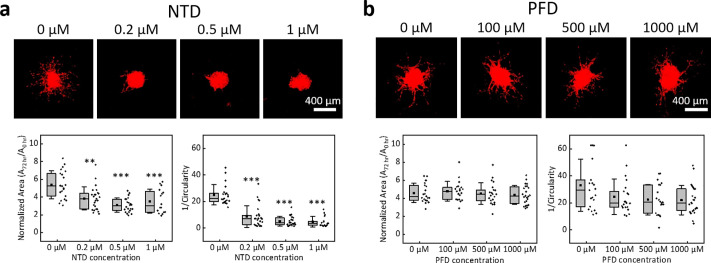
Effects of anti-fibrotic drugs on the growth and invasiveness of A549 cell spheroids in fibrin/Matrigel co-cultured with MRC-5 fibroblasts. (a) Top: confocal fluorescence projection images of typical A549 cell spheroids co-cultured with fibroblasts in fibrin/Matrigel treated with various concentrations of NTD for 72 h. Bottom: projection areas and 1/Circularity values of A549 cell spheroids under the treatment of NTD treatment. (b) Results with the PFD treatment. The data were obtained from three independent experiments. Each dot represents one spheroid. ***, *P* < 0.001; **, *P* < 0.01 in comparison with the group without NTD treatment (Dunn's post hoc test).

The results in Fig. 2 suggest that fibroblasts might enhance tumor growth and cancer cell invasion in fibrin/Matrigel. Therefore, to know how the anti-fibrotic drugs affected this cancer cell–fibroblast co-culture system, we used various concentrations of NTD and PFD to treat cancer cell spheroids co-cultured with fibroblasts (concentration: 1 × 10^6^ ml^−1^) in fibrin/Matrigel and measured the area and 1/*C* values of the spheroids. The concentration ranges of NTD and PFD were used according to the report of Knüppel *et al.*^40^ The results in Fig. 3(a) show that the treatment of 0.5 or 1.0 *μ*M NTD for 72 h significantly reduced both the area and 1/*C* value of cancer cell spheroids (*P* < 0.001). However, the area and 1/*C* value were still larger than those in the monoculture condition [Figs. 2(c) and 2(d)]. In contrast, 100, 500, and 1000 *μ*M PFD showed no effect on the spheroid areas and 1/*C* values, as shown in Fig. 3(b).

The cell viability (alamarBlue) assays confirmed that both 0.5 *μ*M NTD and 1000 *μ*M PFD did not reduce the viability of MRC-5 fibroblasts in 72 h (Fig. S3 in supplementary material). On the other hand, 50 or 100 *μ*M CDDP significantly diminished the viability of both A549 cells and MRC-5 fibroblasts. Therefore, it would be interesting to know how the combination of NTD or PFD with CDDP affected spheroid growth and cancer cell invasion in the fibrin/Matrigel system.

### Efficacy of CDDP plus anti-fibrotic drugs on cancer cell spheroids with and without fibroblast co-culture

**FIG. 4.**
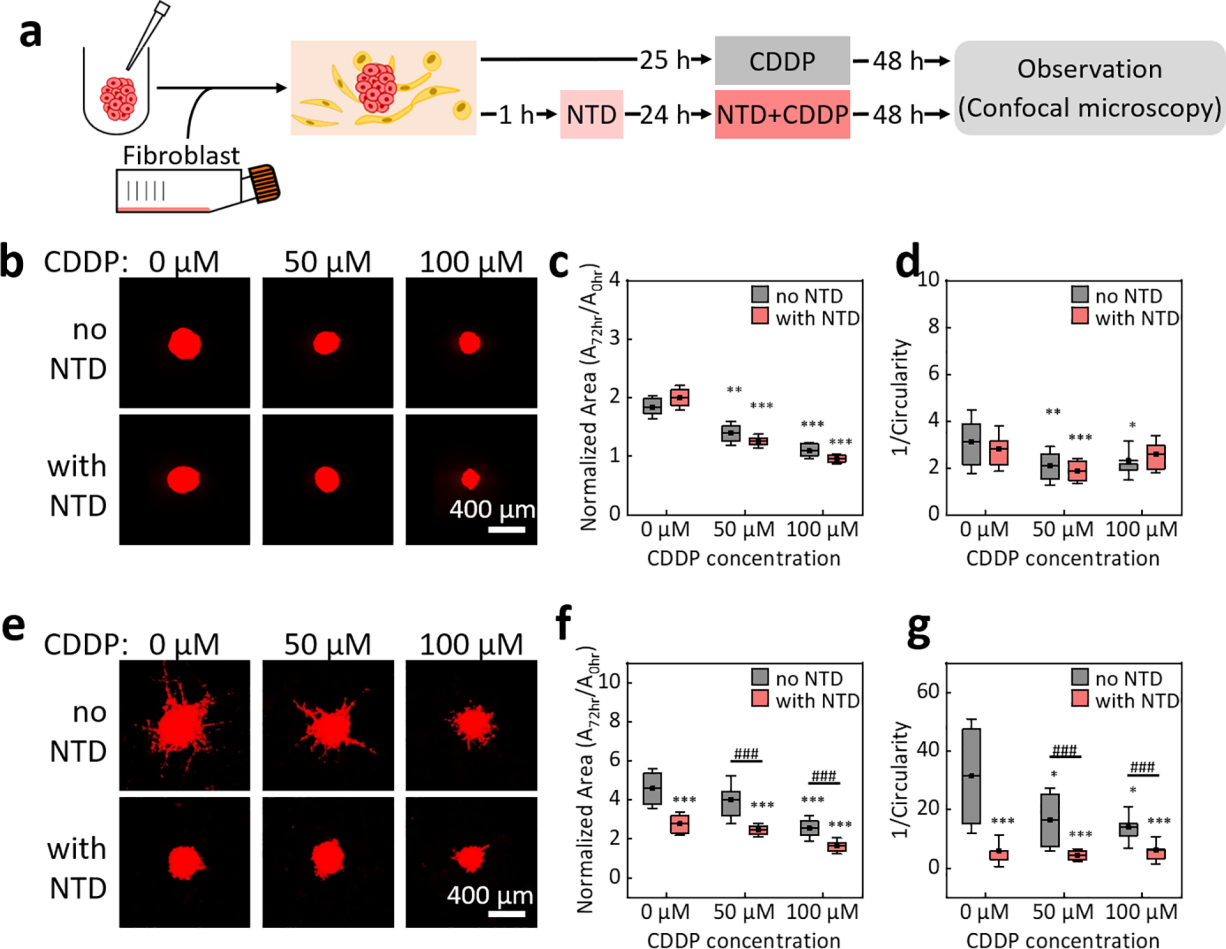
Effects of CDDP and CDDP plus NTD on A549 cell spheroids in fibrin/Matrigel. (a) Experimental procedures. (b) Confocal fluorescence projection images of typical A549 cell spheroids without fibroblasts under the treatments of CDDP with various concentrations for 72 h (top) and with the combination of 0.5 *μ*M NTD (bottom). (c) Projection areas and (d) 1/Circularity values of A549 cell spheroids under the treatment of CDDP and CDDP+NTD. (e)–(g) are the same as (b)–(d) but with fibroblast co-culture. ***, *P* < 0.001; **, *P* < 0.01; *, and *P* < 0.05 in comparison with the group without any treatment. ###, *P* < 0.001 between the two groups as indicated. The significance of differences was checked with the Kruskal–Wallis test and Dunn's post hoc test.

We then evaluated the efficacy of CDDP in combination with NTD or PFD on A549 cell spheroids in fibrin/Matrigel with and without fibroblasts. The duration of anti-fibrotic drug pretreatment was 24 h, after which CDDP was applied for another 48 h [Fig. 4(a)]. All the results were normalized to those obtained right before the pretreatment (0 h) on the same spheroid. The data in Figs. 4(b)–4(d) show that without any treatment, the areas of monocultured A549 cell spheroid increased by nearly 90% after 72 h of culture. The treatment of 100 *μ*M CDDP inhibited the growth of spheroids almost completely in the same period. The addition of 0.5 *μ*M NTD did not show a measurable change in the results of CDDP treatment. The 1/*C* values of monocultured cancer cell spheroids were mostly between 2 and 4, and CDDP treatment reduced the 1/*C* values to ∼2. These results suggest that the invasion of A549 cancer cells was not obvious in fibrin/Matrigel without fibroblasts. The addition of NTD did not affect the 1/*C* values for 50-*μ*M or 100-*μ*M CDDP treatments. We repeated the above experiments using CDDP and PFD, and the data in Figs. S4(a)–S4(c) in the supplementary material show that the addition of 1000 *μ*M PFD did not change the effects of CDDP on the growth and the invasiveness of monocultured A549 cell spheroids.

Next, we evaluated the efficacy of CDDP in combination with NTD or PFD on A549 cancer cell spheroids co-cultured with fibroblasts. The concentration of fibroblasts in the hydrogel was 1 × 10^6^ ml^−1^. Figure 4(e) shows that the addition of 0.5 *μ*M NTD significantly reduced the fibroblast-assisted invasion of cancer cells in the co-culture system. According to the data in Figs. 4(f) and 4(g), the effects of 0.5 *μ*M NTD without CDDP on the spheroid area and 1/*C* value were close to those of 100 *μ*M CDDP without NTD in the co-culture system. The treatment of 0.5 *μ*M NTD + 50 *μ*M CDDP suppressed the spheroid area and 1/*C* value to those under the 100 *μ*M CDDP treatment, suggesting that the combination of NTD might lead to a reduction in the CDDP dosage while preserving the antitumor effects. Moreover, the addition of 0.5 *μ*M NTD to 50 *μ*M or 100 *μ*M CDDP reduced the spheroid areas and 1/*C* values more than CDDP alone.

We also evaluated the effect of the combination of PFD and CDDP on A549 cell spheroids co-cultured with fibroblasts. Figures S4(d)–S4(f) in the supplementary material show that the addition of 1000 *μ*M PFD to 50 *μ*M or 100 *μ*M CDDP did not exhibit observable effects on the spheroid area and 1/*C* values. Therefore, we hypothesized that the addition of 1000 *μ*M PFD did not improve the effect of CDDP treatment on the co-culture system.

Although there have been many studies using 2D culture to explore the combined effects of anti-fibrotic drugs with anticancer drugs on CAFs and cancer cells,^41–44^ no work directly compared NTD and PFD on the proliferation and invasion of cancer cells, even in a 2D co-culture system. In the present work, for monocultured A549 cell spheroids in fibrin/Matrigel, the effects of CDDP and CDDP plus NTD or PFD on reducing the spheroid areas did not differ significantly. However, with the presence of fibroblasts, the combination of 50 *μ*M CDDP and 0.5 *μ*M NTD could achieve a size-reduction effect similar to that of 100 *μ*M CDDP alongside an even better invasion-suppression effect than that of 100 *μ*M CDDP. In contrast, the combination of CDDP and 1000 *μ*M PFD did not show any observable variations in the effects of CDDP. This result is different from that obtained with the combination of 10 *μ*M CDDP and 2.7 mM PFD in a conventional 2D co-culture system, wherein the combined treatment did increase the death ratio of cells treated with CDDP alone.^41^ Another study using A549 cell spheroids co-cultured with fibroblasts in 3D collagen gels suggested that 2.7 mM PFD could reduce the expression of α-smooth muscle actin in fibroblasts.^45^ However, in both studies, the effects of PFD on cancer cell invasiveness were not reported. Fujiwara *et al.* showed that in 2D culture PFD alone could suppress fibroblast activities and epithelial to mesenchymal transition of A549 cancer cells induced by the fibroblast conditioned medium.^46^ However, because of the high concentration of PFD (2.7 mM), the invasion and growth of A549 cancer cells were inhibited even without the fibroblast conditioned medium. We hypothesized that in our experiments, the addition of 1000 *μ*M PFD might have only a negligible effect on the interactions between cancer cells and fibroblasts, even under the treatment of 100 *μ*M CDDP. Considering that the measured PFD concentrations in the blood of adult patients were less than 220 *μ*M,^47^ our results could be closer to the conditions in living tissues.

### Efficacy of anti-fibrotic drugs on fibroblasts in fibrin/Matrigel

The results in Fig. 4 indicate that for the monocultured A549 cell spheroids, the addition of anti-fibrotic drugs did not change the efficacy of CDDP on spheroid growth and cancer cell invasion. In the co-culture system, the anti-fibrotic drug, NTD, increased the efficacy of CDDP, but PFD did not show a similar effect. Because the co-culture of fibroblasts significantly enhanced spheroid growth and cancer cell invasion (Fig. 2), we assumed that NTD could suppress the activities of fibroblasts in the co-culture system more significantly than PFD. Moreover, the data in Fig. 4(g) suggest that 0.5 *μ*M NTD could reduce the 1/*C* value more than 50 *μ*M CDDP. Therefore, comparing the effects of NTD and PFD on fibroblasts in fibrin/Matrigel might be useful in revealing more details about the results shown in Fig. 4.

**FIG. 5.**
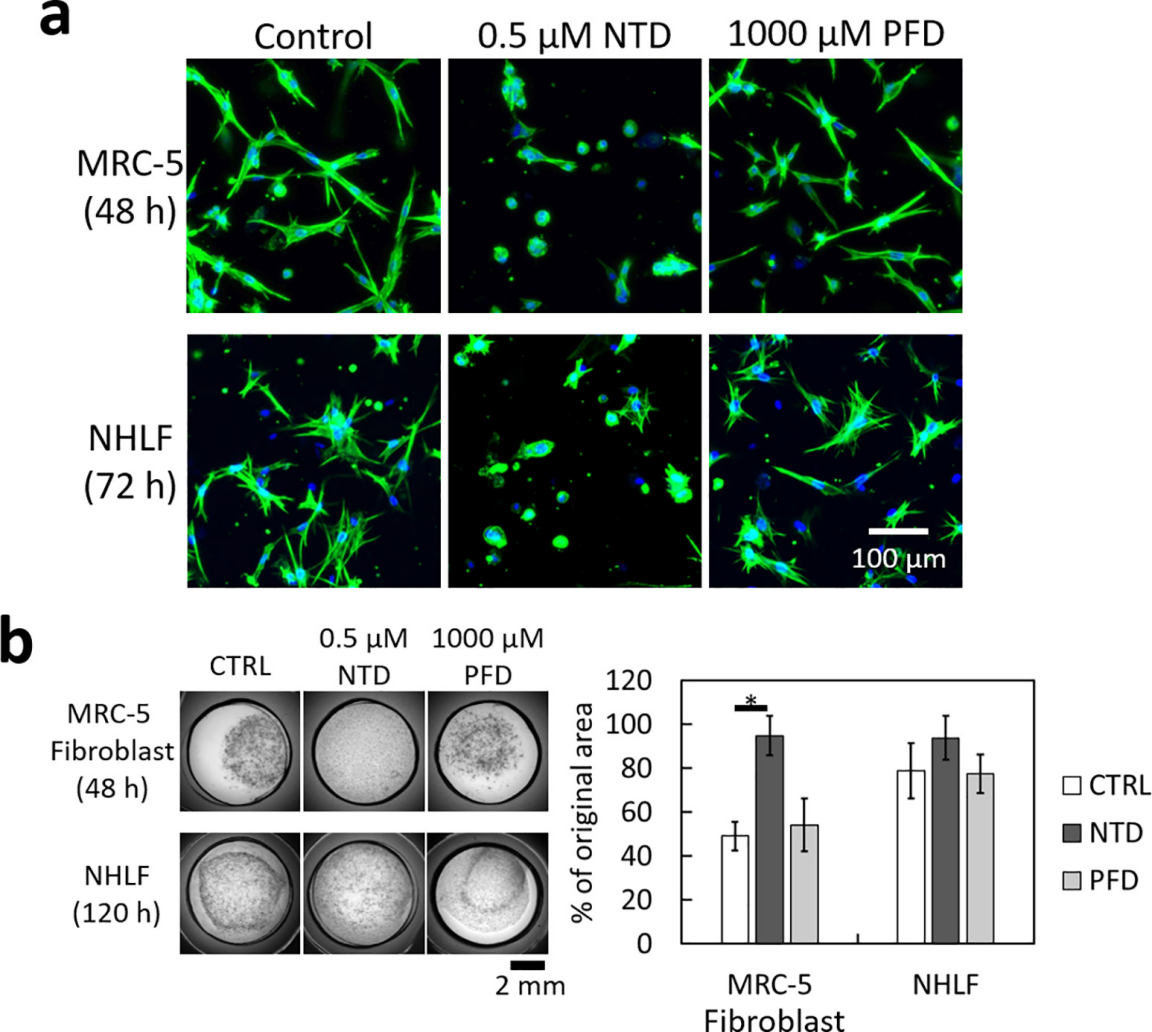
Effects of anti-fibrotic drugs on the activities of fibroblasts. (a) Confocal fluorescence projection images of fixed MRC-5 fibroblasts and normal human lung fibroblasts (NHLFs) in fibrin/Matrigel. The fibroblasts were in the medium conditioned by A549 cells and treated by NTD or PFD. Only NTD treatment changed cell morphology. Green, F-actin labeled with phalloidin-FITC. Blue, cell nuclei labeled with DAPI. (b) Area variations of fibrin/Matrigel laden with fibroblasts in the A549 cell-conditioned medium. With the MRC-5 fibroblasts, the hydrogel area was decreased by ∼50% after 48 h of culture. With the NHLFs, the hydrogel area was decreased by ∼20% after 120 h of culture. The 0.5 *μ*M NTD treatment impeded the area shrinkage caused by both types of fibroblasts. In contrast, the 1000 *μ*M PFD treatment did not show an observable effect. The data presented are from three independent experiments. *, *P* < 0.05 (Dunn's post hoc test).

We used confocal microscopy to observe the morphological variations of MRC-5 fibroblasts in fibrin/Matrigel. The medium was conditioned by A549 cells (2 × 10^5^ ml^−1^, 24 h of culture). Figure 5(a) shows that the 0.5 *μ*M NTD treatment for 48 h substantially changed the morphology of fibroblasts, and the actin filaments did not increase in length as those in fibroblasts treated with PFD. As further verification of the different effects between NTD and PFD on fibroblasts, we also treated normal human lung fibroblasts (NHLFs) in fibrin/Matrigel. Because of the slower response of NHLFs, the duration of treatment was extended to 72 h. The effects of NTD and PFD on NHLFs were similar to those on MRC-5 fibroblasts [Fig. 5(a)]. By combining the observation in Fig. 5(a) with the results in Fig. 4, we hypothesized that NTD treatment reduced the motility of fibroblasts in fibrin/Matrigel effectively, even without CDDP. Therefore, the suppression of cancer cell invasion by NTD might be mostly caused by the suppression of fibroblast activities.

As another assessment of fibroblasts' activities, we measured the decrease in hydrogel areas caused by the fibroblast contraction^48^ with and without NTD or PFD treatment. Figure 5(b) shows that without anti-fibrotic drugs, both MRC-5 fibroblasts (after 48 h of culture) and NHLFs (after 120 h of culture) in the A549 cell-conditioned medium induced area shrinkage of fibrin/Matrigel, but the contraction caused by NHLFs (∼20%) was much smaller than that caused by MRC-5 fibroblasts (∼50%). The 0.5 *μ*M NTD treatment impeded the area shrinkage of fibroblast-laden fibrin/Matrigel. In contrast, the 1000-*μ*M PFD treatment did not exhibit a similar effect. This result suggests that the contraction of fibroblasts in fibrin/Matrigel might be suppressed by NTD but not by PFD.

The shrinkage of hydrogels caused by fibroblasts inside could lead to the underestimation of the growth and invasion of cancer cells. Therefore, with the co-culture of fibroblasts, the tumor growth and cancer cell invasion of the control and PFD-treated groups could be higher than what we measured. In contrast, for the NTD-treated group, the evaluations of spheroid areas and invasiveness should be more accurate. In other words, the efficacy of NTD in the suppression of tumor growth and cancer cell invasion in the co-culture system could be more evident than what we observed in Figs. 4(e)–4(g).

To further determine the effects of anti-fibrotic drugs on fibroblasts in 3D culture, we performed RNA sequencing on MRC-5 fibroblasts in fibrin/Matrigel treated with 0.5 *μ*M NTD or 1000 *μ*M PFD for 48 h. The fibroblasts were in the medium conditioned by A549 cells (2 × 10^5^ ml^−1^, 24 h of culture) to mimic the co-culture condition. We identified the expression of four genes relevant to cell adhesion, invasion, or ECM degradation that were reduced by NTD but increased or unaffected by PFD. The target genes were selected with two criteria: (1) the log_2_ (fold change between NTD and control groups) value was smaller than –0.42, which meant that NTD treatment reduced this gene expression by more than 25% compared with that in the control group, and (2) the log_2_(fold change between NTD and PFD groups) value was smaller than −1.0, which meant that this gene expression under NTD treatment was less than 50% compared with that under PFD treatment. The log_2_ values of fold changes between NTD and control groups, and between NTD and PFD groups, of these four genes (*AFAP1L2, MMP16, ITGA8*, and *POSTN*) are listed in Table I. As a comparison, the log_2_(fold change between NTD and PFD groups) values of common genes in CAFs, *ACTA2* (α-smooth muscle actin), *COL1A2* (collagen I), *FN1* (fibronectin), and *VIM* (vimentin) were between –0.29 and 0.82, indicating only small differences in the expression of these genes between the fibroblasts treated by NTD and those by PFD.

**TABLE I.** Four genes relevant to cell adhesion, invasion, or ECM degradation in MRC-5 fibroblasts cultured in fibrin/Matrigel, whose expression were reduced by NTD treatment for more than 25% compared with those in the control group. The expression of these genes under NTD treatment was less than 50% compared with that under PFD treatment.

Gene symbol	Protein description	Log_2_ (fold change between NTD and control groups)	Log_2_ (fold change between NTD and PFD groups)
*AFAP1L2 (XB130)*	Actin filament-associated protein 1 like 2	−1.57	−1.37
*MMP16*	Matrix metallopeptidase 16	−1.30	−3.35
*ITGA8*	Integrin subunit alpha 8	−1.08	−2.85
*POSTN*	Periostin	−0.55	−3.55

Among the proteins encoded by the four genes listed in Table I, actin filament-associated protein 1 like 2 (encoded by *AFAP1L2*, also known as *XB130*) is known to be involved in the formation of lamellipodia, and the downregulation of *AFAP1L2* could reduce cell migration and invasion.^49^ The reduced expression of *MMP16* by NTD could lower the invasion capability into the ECM.^50^ The reduction of *ITGA8* expression could decrease cell adhesion on ECM,^51^ and therefore, the fibroblasts were not able to extend into the spindle shape in the fibrin/Matrigel [Fig. 5(a)]. Meanwhile, decreased cell adhesion in the ECM could also reduce the contraction force on the hydrogel exerted by fibroblasts, which is in line with the results shown in Fig. 5(b). Finally, NTD could also reduce periostin (encoded by *POSTN*), a cell-adhesion protein that is highly expressed in various types of cancers and positively correlated with cancer invasion and metastasis.^52^ Periostin secreted by activated fibroblasts in tissues of idiopathic pulmonary fibrosis could increase the proliferation of lung cancer cells.^53^

Although to explore the detailed signaling pathways of proteins encoded by these genes under NTD or PFD treatment will require several additional experiments, RNA sequencing data suggest that the effects of NTD on CAFs could be more suppressive than PFD in terms of cell adhesion, invasion, or ECM degradation. This is consistent with the results of our 3D co-culture experiments. In addition, because NTD could reduce the expression of genes related to cell adhesion or migration, such as *ITGA8* and *AFAP1L2*, and those genes controlling secretive proteins, such as *MMP16* and *POSTN*, the physical and biochemical mechanisms must be taken into account together to correctly explain the effects of anti-fibrotic drugs on the cancer cell–fibroblast co-culture system.

NTD has been confirmed to improve the effects of anticancer drugs. For example, NTD combined with docetaxel was suggested to be second-line therapy for patients with non-small-cell lung cancer who have been treated with platinum-based drugs.^54^ Recently, NTD combined with carboplatin plus nab-paclitaxel was found to improve the overall survival in lung cancer patients with idiopathic pulmonary fibrosis.^55^ In an animal study, NTD and an anti-VEGF/Ang2 nanobody were used together to reduce the brain metastases of lung cancer cells.^56^ The results of these clinical trials and the *in vivo* test are in agreement with that of our 3D co-culture experiments, supporting that NTD could improve the efficacy of anticancer drugs. On the other hand, we have not found any published results of clinical trials about the combined effect of PFD and anticancer drugs on lung cancer. Animal studies showed that PFD could improve the antitumor effects of cisplatin^41^ or carboplatin.^57^ However, the dose of PFD used in these two works was 200 mg/kg body weight per day, which is about five times that recommended for human patients.^58^ In a follow-up work, Fujiwara *et al.* demonstrated that PFD of the same dose alone could reduce the subcutaneous tumor volume of A549 cells.^46^ Increasing the daily dose to 250 or 500 mg/kg, Marwitz *et al.* also showed that PFD reduced the tumor growth of subcutaneously transplanted Lewis lung carcinoma cells.^59^ The efficacy of high-dose PFD in animal studies should be valid, but the dosage might be much higher than that clinically applicable and tolerable in humans. In contrast, with the 3D co-culture model, we can control the drug concentrations more precisely, and therefore, the results could be more relevant to future clinical applications.

## CONCLUSION

Owing to the versatile roles of CAFs in the TME, various reagents have been proposed to target CAFs in cancer therapy.^60^ Anti-fibrotic drugs could also be considered active components in anticancer therapy because they may modulate the ECM and CAF activities. Gabasa *et al.* reported that NTD could inhibit the interactions between lung cancer cells and CAFs.^43^ Nonetheless, different anti-fibrotic drugs may change CAF activities via different mechanisms. For example, both NTD and PFD reduced the expression of collagen V in fibroblasts derived from patients with idiopathic pulmonary fibrosis; however, NTD also suppressed the expression of collagen I, fibronectin, and FKBP10.^40^ Therefore, a reliable 3D co-culture system of cancer cells and fibroblasts is highly desirable for evaluating the combined effects of anticancer drugs and various ECM-targeting drugs.^1,2^

In the present study, we used the mixture of fibrin and Matrigel to construct a 3D co-culture environment for cancer cell spheroids and fibroblasts. The images acquired by FIB-SEM and the stiffness measured by AFM indicated that fibrin provided additional fibrillar structures and increased the stiffness of the hydrogel. The morphology of fibroblasts in fibrin or fibrin/Matrigel was spindle shape, in contrast to the spherical morphology in Matrigel. We used this 3D co-culture system to evaluate the effects of CDDP in combination with anti-fibrotic drugs, NTD or PFD, on the growth and invasion of lung cancer cell spheroids. The combination of 50 *μ*M CDDP and 0.5 *μ*M NTD exhibited a size-reduction effect similar to that of 100 *μ*M CDDP and a better invasion-suppressive effect. In contrast, the addition of 1000 *μ*M PFD exhibited negligible variations in the efficacy of CDDP. RNA sequencing data suggested that NTD could have a higher ability to reduce the expression of four genes associated with cell adhesion, invasion, and ECM degradation than PFD. This fibrin/Matrigel co-culture system could be useful for evaluating the effects of drug combinations on various types of tumors related to tissue fibrosis, such as lung cancer, liver cancer, and oral cancer. Further studies on drug combination evaluations with patient-derived tissues cultured in fibrin/Matrigel are required.

## METHODS

### The 3D co-culture model

We used the A549 (RRID:CVCL_0023) human lung adenocarcinoma cell line, MRC-5 (RRID:CVCL_0440) human lung fibroblast cell line (both from Bioresource Collection and Research Center, Hsinchu, Taiwan), and NHLF cell line (CC-2512, Lonza, Basel, Switzerland) in this study. A549 cancer cells were cultured in the F12K medium, whereas MRC-5 fibroblasts were cultured in MEM-α. The culture medium for NHLFs was FGM^TM^-2 BulletKit^TM^ Growth Media (CC-3132, Lonza). The spheroid formation process is as follows: on the first day, A549 cells were stained with 2.5 *μ*M CellTracker™ Red CMTPX Dye (C34552, Thermo Fisher Scientific) for 30 min and then washed with phosphate-buffered saline (PBS). A549 cells were seeded onto ultra-low-attachment 96-well plates (7007, Corning) filled with MEM-α:F12K medium (1:1 v/v) and 10% FBS at a density of ∼1000 cells/well. Cancer cells in each well spontaneously aggregated into a spheroid after ∼24 h. On the second day, the cancer cell spheroid and fibroblasts were re-suspended simultaneously in a hydrogel mixture containing 5 mg/mL fibrinogen (F8630, Sigma-Aldrich) and 3 mg/ml Matrigel^®^ (356231, Corning) in MEM-α:F12K medium. We added 2.5 U/ml thrombin (T7513, Sigma-Aldrich) into this cell-hydrogel mixture and kept it at 37 °C for an hour to polymerize fibrinogen into fibrin.

For the drug combination tests, all the chemicals were kept on ice before being administered. Either nintedanib (NTD, SC-482704, Santa Cruz Biotechnology) or pirfenidone (PFD, P2116, Sigma-Aldrich) was applied 24 h before cisplatin treatment (CDDP, 232120, Sigma-Aldrich) to ensure its effects on fibroblasts.

### FIB-SEM imaging of the hydrogels

FIB-SEM imaging was performed using FEI Helios NanoLab 660 (Thermo Fisher Scientific) equipped with a cryo-preparation system (PP3010, Quorum). The sample preparation procedures were as follows: hydrogel samples were rinsed with PBS and then fixed with 4% glutaraldehyde at 4 °C overnight and then post-fixed with 1% OsO_4_ in ddH_2_O for 1 h. The samples were further washed with PBS three times. The hydrated samples were carefully mounted and quickly frozen by dipping them into liquid nitrogen. The frozen samples were transferred onto a cryo-stage (−140 °C) and fractured with a knife in the Quorum chamber. Thereafter, the temperature of the stage was set to −85 °C and then gradually increased to −50 °C (5 °C/min). The temperature was maintained at −50 °C for five more minutes, after which it was reduced to −140 °C. The sublimed samples were sputter-coated with platinum in the SEM (10 mA, 60 s) to increase the conductivity. The images were acquired with a 50-pA beam current at a 5-kV acceleration voltage using the concentric backscatter detector of the SEM.

To calculate the average pore sizes and number densities of hydrogels in the SEM images, we used the Analyze Particle function in ImageJ. For each type of hydrogel, we independently prepared two samples and acquired four SEM images on each sample. The field of view of each image was 31.5 × 22.5 *μ*m^2^.

### Evaluation of the drug efficacy

We dissolved CDDP in PBS containing 140 mM NaCl. Anti-fibrotic drugs, NTD and PFD, were dissolved in dimethyl sulfoxide. CDDP solution (3.3 mM) and PFD solution (1.0 M) were stored at 4 °C, whereas NTD solution (20 mM) was stored at −20 °C. All the drugs were used within less than 2 weeks after preparation.

We used the projection area of a spheroid to quantify tumor enlargement. The projection area was measured using a confocal microscope (LSM 880, Carl Zeiss Microscopy GmbH) with a 10×, 0.45 NA objective lens. We captured the optically sectioned images of a spheroid at a 20-*μ*m separation along the z-axis to construct a 3D image stack and then used the Maximum Intensity Projection function of ImageJ to obtain the projection area of the spheroid. The area of a spheroid after 72 h of drug treatment was normalized to the initial value as the evaluation of the drug efficacy on the spheroid size.

To quantify the cancer cell invasion capability in the 3D culture, we used the inverse of the spheroid circularity to estimate the irregularity of the spheroid contour. The circularity *C* was defined as *C* = 4π*A*/*P*^2^, where *A* and *P* were the projection area and perimeter of the spheroid, respectively. *C* is equal to one for a perfect circle. If the shape of a spheroid was irregular, the value of *P* would increase, leading to a higher value of 1/*C*. Therefore, a higher 1/*C* value indicated the condition with more cells invading the surrounding hydrogel. Thus, a spheroid with a larger projection area and higher 1/*C* value could be considered more malignant in this 3D culture model.

### Fluorescence imaging of F-actin in fibroblasts

We labeled the F-actin in fibroblasts with Alexa Fluor™ 488 Phalloidin (A12379, Thermo Fisher Scientific). In brief, fibroblast-embedded hydrogels were fixed with 4% paraformaldehyde/PBS at 4 °C overnight, followed by triple PBS washes. The samples were permeabilized with 0.1% Trion X-100 for 30 min, followed by triple PBS washes. The samples were blocked in 1% bovine serum albumin for 1 h at room temperature and then incubated in Alexa Fluor™ 488 Phalloidin for 1 h. Subsequently, the samples were washed with PBS and stained with 1 *μ*g/ml DAPI solution (62248, Thermo Fisher Scientific). The fluorescence images were captured using a Zeiss LSM 880 confocal microscope.

### Hydrogel area measurement

We seeded MRC-5 fibroblasts at a concentration of 1 × 10^6^ ml^−1^ into the fibrin/Matrigel containing the medium conditioned by A549 cells. The concentration of NHLFs was 2 × 10^6^ ml^−1^. After 1 h of culture, we added the anti-fibrotic drug into the hydrogel and then captured the images of the hydrogels after another 48 h (for MRC-5 fibroblasts) or 120 h (for NHLFs) of culture using a 2×, 0.10 NA objective lens. We manually marked the hydrogel boundary in the images and measured the hydrogel area using ImageJ.

### RNA sequencing

We used purified RNA for the preparation of the sequencing library using the TruSeq Stranded mRNA Library Prep Kit (Illumina, San Diego, CA, USA) per the manufacturer's protocol. In brief, mRNA was purified from total RNA (1 *μ*g) by oligo(dT)-coupled magnetic beads and fragmented into small pieces at high temperatures. The first-strand cDNA was synthesized using reverse transcriptase and random primers. After the generation of double-strand cDNA and adenylation on 3′ ends of DNA fragments, the adaptors were ligated and purified using the AMPure XP system (Beckman Coulter, Beverly, USA). The quality of the libraries was assessed using the Qsep 400 system. The qualified libraries were then sequenced using an Illumina NovaSeq 6000 platform with 150-bp paired-end reads generated by Genomics, BioSci & Tech Co. (New Taipei City, Taiwan).

The data were collected from two independent experiments. Low-quality bases and sequences from adapters in the raw data were removed using fastp (version 0.20.0).^61^ The filtered reads were aligned to the reference genomes using HISAT2 (version 2.1.0).^62^ The software, featureCounts (v2.0.1),^63^ in the Subread package, was used for the quantification of gene abundance. Differentially expressed genes were identified by DESeq2 (version 1.28.0).^64^

### Statistical analysis

Data distributions were examined using the Shapiro–Wilk test. For normally distributed data, the significance of the difference was evaluated using the ANOVA for grouped data with Tukey's post hoc test. If the distribution was non-normal, the significance of the difference was evaluated using the Mann–Whitney U test for a pair of data or the Kruskal–Wallis test for grouped data with Dunn's post hoc test.

## SUPPLEMENTARY MATERIAL

See the supplementary material for details of (1) AFM measurement of hydrogel stiffness, (2) monocultured cell viability and invasion in hydrogels, (3) monocultured cell viability in CDDP, NTD, and PFD of various concentrations, and (4) combined effects of CDDP and PFD on A549 cell spheroids with or without the co-culture of fibroblasts.

## Data Availability

The data that support the findings of this study are available within the article and its supplementary material.
